# Case Report: Diagnostic challenges in Fabry disease: misinterpreted obstructive hypertrophic cardiomyopathy and the role of enzyme replacement therapy

**DOI:** 10.3389/fcvm.2024.1479374

**Published:** 2024-11-22

**Authors:** Weili Cheng, Mingqiang Ao, Dinghu Xu, Yuqing Zhang, Qin Tao

**Affiliations:** ^1^ Department of Cardiology, The Affiliated Jiangning Hospital of Nanjing Medical University, Nanjing, China; ^2^ Department of Radiology, The Affiliated Jiangning Hospital of Nanjing Medical University, Nanjing, Jiangsu, China

**Keywords:** case report, Fabry disease, GLA gene, enzyme replacement therapy, prognosis

## Abstract

Fabry disease (FD) is a rare X-linked inherited lysosomal storage disorder. The abnormal accumulation of metabolic substrates induces inflammation and fibrosis in cells, resulting in organ dysfunction. The clinical manifestations of FD are diverse and non-specific. In the present study, we report a case initially treated as obstructive hypertrophic cardiomyopathy for several years, which was finally identified as FD through whole-exome sequencing (WES). The patient, diagnosed with obstructive hypertrophic cardiomyopathy, underwent left ventricular outflow tract surgery before visiting our hospital. WES was proposed by our cardiomyopathy center and, unexpectedly, a mutation [c.595T>C (p.Val199Met)] in exon 4 of the GLA gene was identified. A subsequent analysis of plasma α-galactosidase and globotriaosylsphingosine levels confirmed the diagnosis of FD. Although enzyme replacement therapy (ERT) was initiated immediately after diagnosis, the patient experienced aortic valve damage and left heart enlargement 2 years later. Subsequently, the patient underwent transcatheter aortic valve replacement. This case implies that FD should be considered a potential cause in patients with unexplained left ventricular hypertrophy. Delayed initiation of ERT may compromise its efficacy.

## Introduction

Fabry disease (FD) is a rare X-linked inherited lysosomal storage disorder characterized by a deficiency of α-galactosidase A (GLA), resulting from mutations in the GLA gene. This deficiency leads to the progressive accumulation of metabolic substrates, particularly globotriaosylceramide and galactosylceramide, within lysosomes throughout the body (1). The aberrant storage of these molecules incites inflammation and fibrosis in cells, ultimately resulting in organ dysfunction (2). The clinical manifestations of FD are diverse and non-specific (3). In male patients with the hemizygous form, multiple systems—including the kidneys, skin, cornea, nervous system, and heart—are typically affected (4). Cardiac abnormalities are common complications of FD, frequently presenting as left ventricular hypertrophy (5). Enzyme replacement therapy (ERT) represents the primary disease-specific treatment; however, the optimal initial time and efficacy of ERT in FD-related cardiomyopathy remain inadequately defined (6). In this report, an atypical variant of FD mimicking obstructive hypertrophic cardiomyopathy is presented. A mutation in the GLA gene, identified through whole-exome sequencing (WES), ultimately unveiled the diagnosis of Fabry disease. Notably, 2 years after the initiation of ERT, the patient developed significant aortic valve damage and heart failure.

## Case description

**Figure 1:**
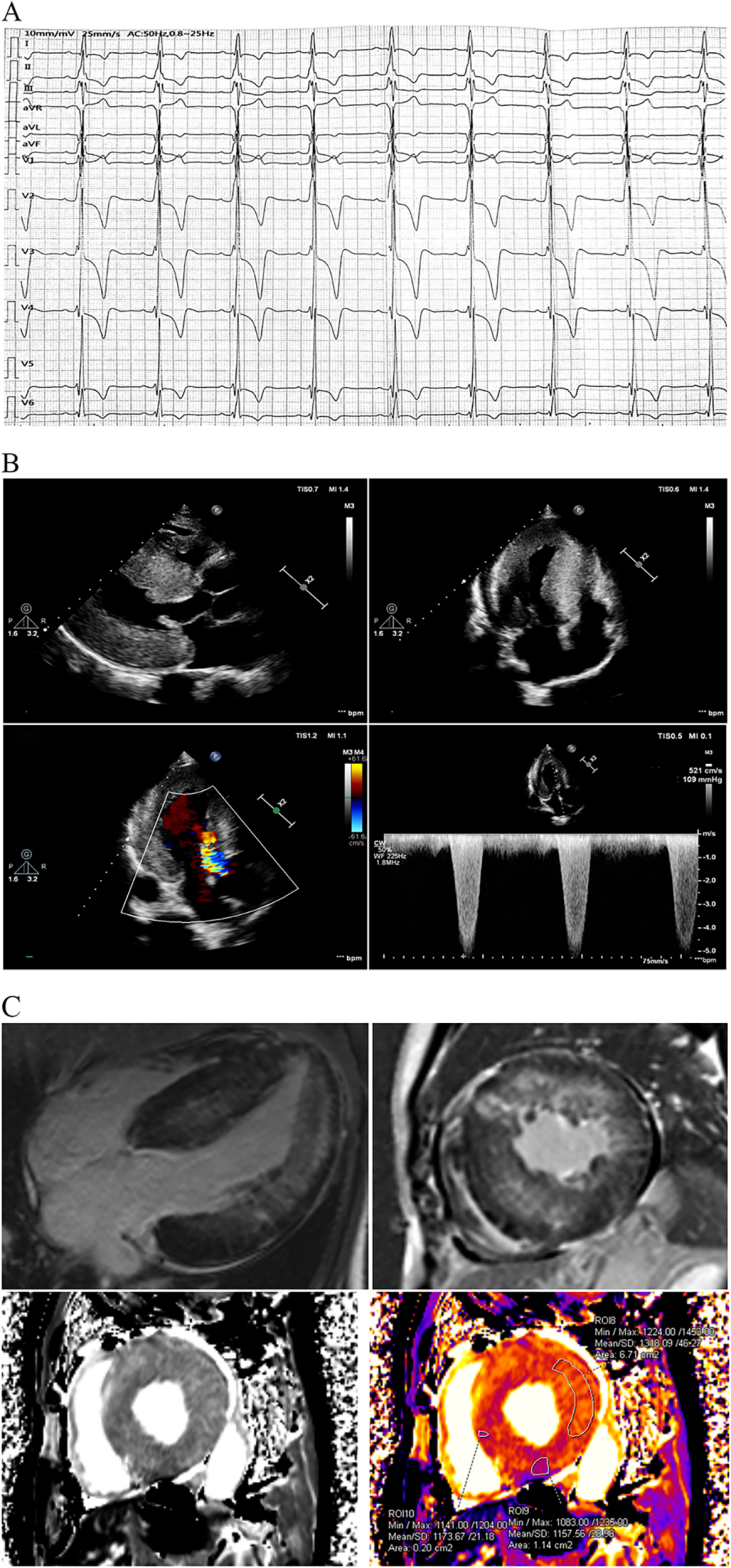
Clinical data at first medical contact. **(A)** The ECG exhibited inverted T-waves with abnormal QRS complexes in nearly all leads, indicating left ventricular hypertrophy. The PR interval measured 202 ms, suggesting that the patient was in an advanced stage of the disease. **(B)** TTE revealed severe concentric left ventricular hypertrophy consistent with obstructive hypertrophic cardiomyopathy, with a left ventricular outflow tract gradient of 109 mmHg at rest. **(C)** CMR using late gadolinium enhancement demonstrated diffuse fibrosis of the left ventricle. Notably, T1 mapping on CMR was not reduced, which contrasts with previous reports. ECG, electrocardiogram; FD, Fabry disease; TTE, transthoracic echocardiography; CMR, cardiac magnetic resonance imaging.

The proband sought medical advice for mild chest discomfort in 2019. Physical examination revealed a grade 4/6 systolic ejection murmur in the third and fourth left intercostal spaces upon cardiac auscultation. The electrocardiogram showed inverted T-waves and abnormal QRS complexes in nearly all leads (Figure 1A). Transthoracic echocardiography (TTE) revealed severe concentric left ventricular hypertrophy consistent with obstructive hypertrophic cardiomyopathy (Figure 1B). The diastolic interventricular septal thickness measured 27 mm, while the maximum thickness at the basal segment was 30 mm. The diastolic left ventricular posterior wall thickness was 24 mm. Systolic anterior motion of the mitral valve was observed, with a left ventricular outflow tract gradient of 109 mmHg measured at rest. Further confirmation of obstructive hypertrophic cardiomyopathy was obtained via cardiac magnetic resonance imaging (CMR). Late gadolinium enhancement revealed diffuse fibrosis of left ventricle without reduction of T1 mapping (Figure 1C). Coronary angiography was performed due to suspected coronary artery disease, revealing mild stenosis of approximately 20% in the proximal left anterior descending artery.

**Figure 2:**
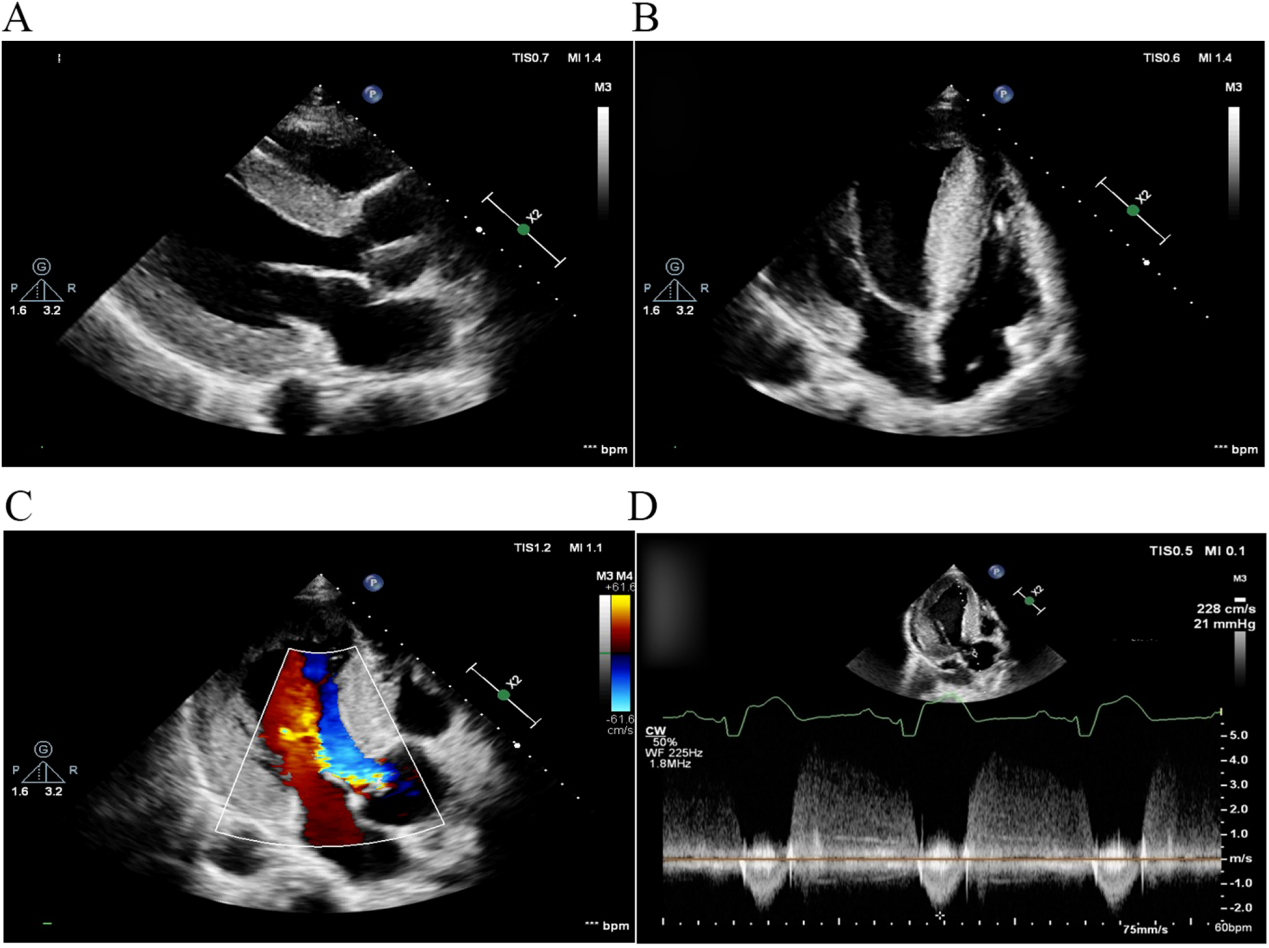
TTE 1 month after left ventricular outflow tract dredging. **(A)** Parasternal long-axis view of the left ventricle and **(B)** apical four-chamber view. The diastolic interventricular septal thickness decreased to 22 mm. **(C)** Color Doppler flow imaging and **(D)** continuous wave Doppler demonstrated that left ventricular outflow tract obstruction was alleviated, with a peak gradient reduced to 21 mmHg. TTE, transthoracic echocardiography.

Two months later, left ventricular outflow tract dredging was performed to alleviate symptoms in the hospital. A permanent pacemaker was implanted due to complete atrioventricular block that occurred as a complication of the surgery. The patient's symptoms resolved, and the resting gradient decreased to 21 mmHg on follow-up TTE 1 month later (Figure 2).

**Figure 3:**
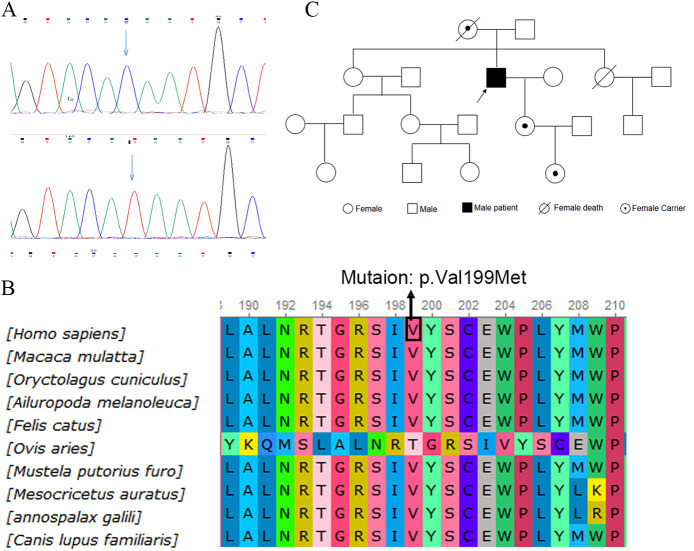
Identification of GLA gene mutation. **(A)** WES identified a mutation [c.595T>C (p.Val199Met)] at exon 4 of the GLA gene, confirmed by Sanger sequencing. **(B)** Alignment of multiple GLA protein sequences in vertebrates. **(C)** Pedigree analysis of the patient's family. WES, whole-exome sequencing.

The patient first presented to our hospital at the age of 53 in 2021 for a routine postoperative check. Diagnosed with obstructive hypertrophic cardiomyopathy, he was enrolled in our study on gene mutations associated with hypertrophic cardiomyopathy. WES identified a mutation [c.595T>C (p.Val199Met)] in exon 4 of the GLA gene, which was confirmed using Sanger sequencing (Figure 3A). A bioinformatics analysis indicated that the mutation is likely damaging (Figure 3B). Considering the association between the GLA gene and Fabry disease, plasma GLA levels and globotriaosylsphingosine (Lyso-GL-3) levels were measured. The GLA level was found to be 0.43 μmol/L/h (reference range = 2.40–17.65 μmol/L/h), while the Lyso-GL-3 level was 12.30 ng/ml (reference range < 1.1 ng/ml). Combining these findings with the diagnostic criteria (7), the underlying cause—Fabry disease—was finally identified. A further pedigree analysis revealed that the patient’s daughter and granddaughter carried heterozygous mutations in the GLA gene (Figure 3C). However, both had normal GLA and Lyso-GL-3 levels. The mutation was not detected in his father or any other unaffected family members. Unfortunately, DNA samples from the patient’s mother were unavailable as she died from cancer at the age of 62 years.

**Figure 4:**
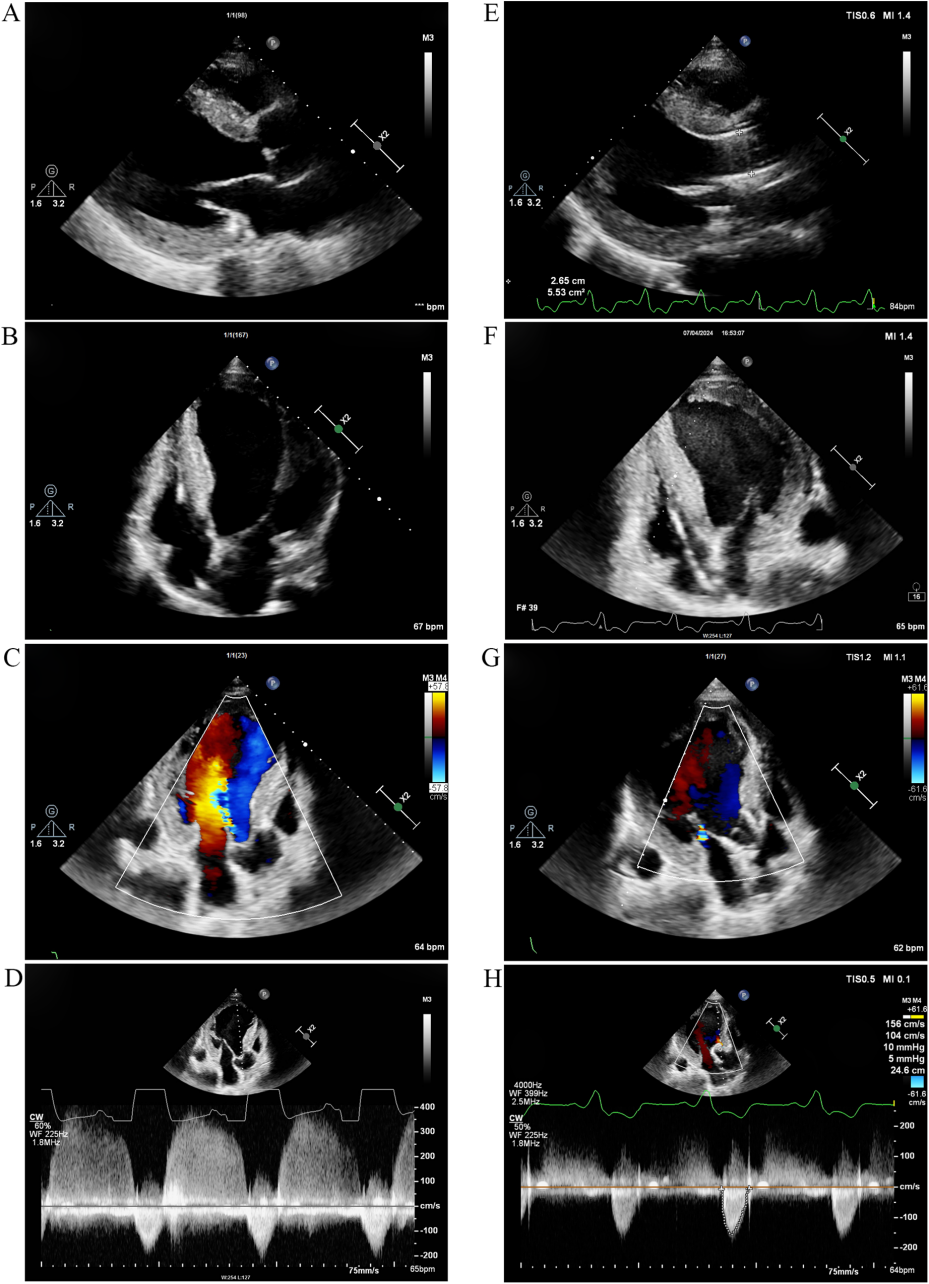
TTE 2 years after ERT and 1 month after TAVR. **(A)** Parasternal long-axis view of the left ventricle and **(B)** Apical four-chamber view 2 years after ERT. The left ventricle was significantly enlarged, and the left ventricular ejection fraction decreased to 27.4%. **(C)** Color Doppler flow imaging and **(D)** continuous wave Doppler 2 years after ERT revealed echo enhancement of the aortic valve and severe aortic regurgitation. **(E)** Parasternal long-axis view and **(F)** apical four-chamber view demonstrated improvement in left ventricular chamber diameter and enhancement of cardiac systolic function 1 month after TAVR. **(G)** Color Doppler flow imaging and **(H)** Continuous wave Doppler indicated that aortic regurgitation improved 1 month after TAVR. TTE, transthoracic echocardiography; ERT, enzyme replacement therapy; TAVR, transcatheter aortic valve replacement.

At the beginning of 2022, ERT was initiated. The patient received *α*-galactosidase at a dosage of 0.2 mg/kg every other week. Unfortunately, in May 2024, he developed symptoms of heart failure, including exertional dyspnea and orthopnea. The N-terminal pro-B-type natriuretic peptide (NT-proBNP) concentration was 1,746 pg/ml. TTE indicated significant left ventricular enlargement, with the left ventricular ejection fraction decreasing to 27.4% (Figures 4A,B). Echo enhancement of the aortic valve was identified via two-dimensional echocardiography and severe aortic regurgitation on Doppler imaging (Figures 4C,D). The patient underwent transcatheter aortic valve replacement (TAVR) at Zhongshan Hospital (Shanghai, China) 1 month later. He had a smooth recovery, and his heart failure symptoms improved significantly postoperatively (Figures 4E–H) (Table 1).

**Table 1:** The alteration of data collected by TTE.

	Before surgery	After surgery	Before TAVR	After TAVR
IVS (mm)	27	22	20	22
LVPW (mm)	24	23	16	16
LVDd (mm)	46	43	72	69
LVDs (mm)	28	30	62	56
LVEF (%)	69.6	62.9	27.4	35.9
LVFS (%)	39.1	30.2	13.1	17.8

TTE, transthoracic echocardiography; TAVR, transcatheter aortic valve replacement; IVS, interventricular septum; LVPW, left ventricular posterior wall; LVDd, left ventricular end diastolic dimension; LVDs, left ventricular end-systolic dimension; LVEF, left ventricular ejection fraction; LVFS, left ventricular fractional shortening.

## Discussion

The estimated incidence of Fabry disease is in the range of 1 in 40,000 to 1 in 117,000 worldwide (2). The clinical manifestations differ significantly between the classic and non-classic forms (8). Male patients with the hemizygous form typically exhibit no detectable GLA activity, with the onset of disease manifestations occurring as early as childhood, characterized by severe acroparesthesias, hypohidrosis, corneal opacities, and angiokeratomas (9). Left ventricular hypertrophy, usually in a non-obstructive form, is one of the common manifestations observed in classic male patients (10).

In this report, we present an atypical variant of FD with manifestations confined exclusively to the heart. The patient's age at onset was significantly later than that of classical cases, and he exhibited no clinical signs typically associated with FD. This lysosomal storage disorder appears to be restricted solely to cardiac involvement. Patients with atypical variants may retain some residual enzyme activity, leading to a more gradual progression of the disease (11). Several atypical variants of FD in hemizygous men have been documented, showing partial deficiency of GLA (12, 13). In our study, residual plasma GLA activity was also detected in the patient. Typically, cardiomyopathy caused by FD is concentric and non-obstructive (14), yet our case developed severe left ventricular outflow tract obstruction, a rare occurrence. Although previous studies have indicated that severe FD cardiomyopathy can evolve into obstructive forms, these cases primarily exhibit midventricular obstruction rather than outflow tract obstruction (15).

Due to the non-specific clinical presentation, diagnosing FD can be particularly challenging. Misdiagnosis is common in cases of FD cardiomyopathy, especially among patients with isolated heart disease. While some researchers have suggested that Fabry's cardiomyopathy can be distinguished by a subendocardial binary appearance on echocardiography (5), it often visually resembles hypertrophic cardiomyopathy. Prior studies have indicated that T1 mapping on CMR in patients with FD typically shows reduced values (16). However, T1 mapping in this case was not diminished, which contradicts previous reports. This inconsistency may be attributed to severe cardiac fibrosis influencing the T1 values, though the underlying mechanisms require further investigation. Unfortunately, myectomy was not performed in this patient, which is a common practice in China. This resulted in the absence of heart tissue for histological examination, thereby missing an opportunity to confirm the diagnosis of FD (17). The patient was initially diagnosed with obstructive hypertrophic cardiomyopathy based on echocardiographic findings before undergoing WES. The genetic basis of FD is well-established, with the GLA gene having been sequenced and numerous mutations identified (5). In this study, a hemizygous mutation in exon 4 of the GLA gene [c.595T>C (p.Val199Met)] was identified, which has been previously reported in the classical phenotype of FD (18). Bioinformatics analysis suggested that this mutation is likely damaging, as indicated by tools such as Polyphen-2, SIFT, and Mutation Taster. The markedly reduced GLA enzyme level and elevated Lyso-GL-3 levels confirmed the diagnosis of FD cardiomyopathy.

Available disease-specific treatments for FD include ERTs, such as α-agalsidase and β-agalsidase, as well as pharmacological chaperone therapy (migalastat) (19). ERT has been the primary treatment for FD for over 20 years worldwide. β-agalsidase received approval at the end of 2019, while α-agalsidase was approved by the National Medical Products Administration in August 2020. The efficacy of ERT in altering the natural history of the disease and improving quality of life is profound (20). However, the optimal timing and effectiveness of ERT in FD-related cardiomyopathy remain unclear. Some studies indicate that early initiation of ERT improves left ventricular hypertrophy and reduces cardiovascular events in pre-hypertrophic and early hypertrophic cardiomyopathy, while the response to ERT in advanced cardiomyopathy of FD is often inadequate (21, 22). In our study, the patient began α-agalsidase therapy 3 years after his initial medical contact. Despite treatment, his cardiomyopathy progressed and he developed severe aortic regurgitation. We hypothesize that this complication may be related to the accumulation of Gb3 and Lyso-Gb3 on the aortic valve; however, the precise mechanism warrants further investigation. Our findings suggest that the response to delayed ERT is poor in advanced cardiomyopathy.

In conclusion, we describe a poor outcome of ERT in an atypical case of FD presenting as obstructive hypertrophic cardiomyopathy. FD should be considered a potential etiology in patients with unexplained left ventricular hypertrophy. Genetic sequencing and identification of disease-causing mutations may provide valuable insights for diagnosing atypical FD. Early initiation of ERT may enhance treatment efficacy.

## Data Availability

The datasets presented in this study can be found in online repositories. The names of the repository/repositories and accession number(s) can be found in the article/Supplementary Material.
